# The Role of the Vagus Nerve in the Migrating Motor Complex and Ghrelin- and Motilin-Induced Gastric Contraction in Suncus

**DOI:** 10.1371/journal.pone.0064777

**Published:** 2013-05-28

**Authors:** Yuki Miyano, Ichiro Sakata, Kayuri Kuroda, Sayaka Aizawa, Toru Tanaka, Takamichi Jogahara, Reiko Kurotani, Takafumi Sakai

**Affiliations:** 1 Area of Regulatory Biology, Division of Life Science, Graduate School of Science and Engineering, Saitama University, Saitama, Japan; 2 Faculty of Pharmaceutical Sciences, Department of Pharmaceutical and Health Sciences, Josai University, Saitama, Japan; 3 Laboratory of Animal Management & Resources, Department of Zoology, Faculty of Science, Okayama University of Science, Okayama, Japan; 4 Graduate School of Science and Engineering, Yamagata University, Yamagata, Japan; INRA, France

## Abstract

The upper gastrointestinal (GI) tract undergoes a temporally coordinated cyclic motor pattern known as the migrating motor complex (MMC) in both dogs and humans during the fasted state. Feeding results in replacement of the MMC by a pattern of noncyclic, intermittent contractile activity termed as postprandial contractions. Although the MMC is known to be stimulated by motilin, recent studies have shown that ghrelin, which is from the same peptide family as motilin, is also involved in the regulation of the MMC. In the present study, we investigated the role of the vagus nerve on gastric motility using conscious suncus—a motilin- and ghrelin-producing small animal. During the fasted state, cyclic MMC comprising phases I, II, and III was observed in both sham-operated and vagotomized suncus; however, the duration and motility index (MI) of phase II was significantly decreased in vagotomized animals. Motilin infusion (50 ng·kg^−1^·min^−1^ for 10 min) during phase I had induced phase III–like contractions in both sham-operated and vagotomized animals. Ghrelin infusion (0.1, 0.3, 1, 3, or 10 µg·kg^−1^·min^−1^ for 10 min) enhanced the amplitude of phase II MMC in sham-operated animals, but not in vagotomized animals. After feeding, phase I was replaced by postprandial contractions, and motilin infusion (50 ng·kg^−1^·min^−1^ for 10 min) did not induce phase III–like contractions in sham-operated suncus. However, in vagotomized suncus, feeding did not evoke postprandial contractions, but exogenous motilin injection strongly induced phase III–like contractions, as noted during the phase I period. Thus, the results indicate that ghrelin stimulates phase II of the MMC via the vagus nerve in suncus. Furthermore, the vagus nerve is essential for initiating postprandial contractions, and inhibition of the phase III–like contractions induced by motilin is highly dependent on the vagus nerve.

## Introduction

Motilin is a 22–amino acid polypeptide that was originally purified from porcine intestinal mucosa in the 1970s and is involved in the regulation of the migrating motor complex (MMC)—particularly in phase III in humans [1] and dogs [2]. Motilin is reported to stimulate gastrointestinal (GI) smooth muscle contraction through a direct or indirect pathway (i.e., the autonomic nervous system [3]–[5], myenteric neurons [3], [6], or smooth muscle [7]–[10]). Several studies have suggested that the main pathway for motilin-induced GI contraction is species-dependent. However, ghrelin, which is from the same peptide family as motilin, is reported to induce premature phase III contractions in the human stomach [11], indicating the involvement of ghrelin in the regulation of the MMC.

In humans and dogs, contractile activity of the upper GI tract differs between the fasted and fed state. The postprandial pattern of intermittent contractions is believed to result in mixing of food in the stomach and pumping of food to the intestines. The gastric emptying of solids is known to be regulated by a coordinated motor pattern between the antrum and the pylorus [12]–[14]; this coordination disappears after vagotomy in rats [15] and is significantly impaired during transient vagal blockade in dogs [16]. Additionally, in humans, cholinergic muscarinic blockade with atropine inhibits the hypoglycemia-induced acceleration of solid gastric emptying [17], suggesting that the vagus nerve has an important role in regulating solid gastric emptying and postprandial motility. In contrast, in the fasted state, the GI tract shows a characteristic contractile pattern termed as the migrating motor complex (MMC). The MMC is believed to have a physiologically important role for the mechanical and chemical cleansing of the empty stomach to prepare for the next meal. The MMC consists of 3 phases: phase I (period of motor quiescence), phase II (period of irregular and low-amplitude contractions), and phase III (period of regular and high-amplitude contractions). In rodents, atropine administration or vagotomy treatment abolishes the occurrence of spontaneous and ghrelin-induced gastric phase III–like contractions, suggesting that gastric phase III–like contractions are mediated by vagal cholinergic pathways [18], [19]. Moreover, MMC in the canine stomach and duodenum does not occur during acute vagal blockade by cervical vagal cooling [20], [21], and the interdigestive pattern is disrupted after total vagotomy in dogs [22]. In contrast, several dog studies have reported that vagal cooling at the diaphragm level had no effect on the presence of gastric phase III activity [23], and vagotomy had little or no effect on the fasted pattern of myoelectric activity in the small intestine [24]. Aeberhard et al. reported that the proximal vagal supply to the dog's stomach is not essential for the control of fasting activity, and disruption of the interdigestive pattern after total vagotomy was likely caused by delayed gastric emptying [25]. In addition, Tanaka et al. [5] reported that vagal innervation to the stomach modulates the pattern of cyclic MMC, but not the presence of gastric interdigestive motility during phase III in dogs. It must therefore be concluded that the vagus nerve is involved in the MMC in mammals.

Studies on the effect of vagotomy on the MMC or motilin- and ghrelin-induced contractions are currently insufficient. One reason for this is that rodents are an inappropriate model for study of the MMC because their gastric contractile patterns differ from that of humans and dogs. Moreover, the motilin gene became a pseudogene in a common ancestor of mice and rats [26]. To overcome this problem, we sought an appropriate small mammal in which to investigate GI motility, and identified the house musk shrew (*Suncus murinus*) as a suitable model. We previously demonstrated the existence of motilin, ghrelin, and their respective receptors in the suncus [27]–[29]. Furthermore, we have studied the contractile properties of the stomach and intestine in conscious, free-moving suncus, and demonstrated that this animal has a similar GI motility pattern to that of humans and dogs [30]. In the present study, we investigated the effect of vagotomy on gastric motor activity in the fasted and fed states, and the reactivity to motilin and ghrelin, using conscious suncus.

## Materials and Methods

### Drugs

Suncus motilin (Scrum Inc., Tokyo, Japan) and human ghrelin (Asubio Pharma Co., Ltd., Hyogo, Japan) were dissolved in 0.1% bovine serum albumin in 1× phosphate-buffered saline (PBS).

### Animals

Experiments were performed using adult male suncus (7–15 weeks) of an outbred KAT strain that was established from a wild population in Kathmandu, Nepal [31]. The animals weighed between 60 and 100 g. They were housed individually in plastic cages that were equipped with an empty can as a nest box, under controlled conditions (23°C±2°C; lights on from 08:00 to 20:00), and with free access to water and commercial trout pellets (No. 5P, Nippon Formula Feed Manufacturing, Yokohama, Japan). The metabolizable energy content of the pellets was 344 kcal/100 g, and the pellets consisted of 54.1% protein, 30.1% carbohydrate, and 15.8% fat. All procedures were approved and performed in accordance with the Saitama University Committee on Animal Research. All efforts were made to minimize animal suffering and to reduce the number of animals used in the experiment.

### Animal preparation

After 3 h of fasting, suncus were anesthetized by an intraperitoneal injection of sodium pentobarbital (50 mg/kg). Through a midline laparotomy, strain-gauge force transducers were implanted on the serosal surface of the gastric body for recording circular muscle contractions. The needle was passed with an attached thread (CR11-60N3; Natsume Seisakusho, Tokyo, Japan) through a hole on both sides of the transducer, which was then sutured to the dorsal portion of the gastric body to prevent adhesion to the liver. The strain-gauge force transducers used in this study were developed in our laboratory, and designed to be smaller than the previously reported equipment [30], [32] by using a smaller strain gauge (KFG-02-120-C1-11; Kyowa Electronic Instruments, Tokyo, Japan). The size of the transducer was 7.5 mm in length and 7.0 mm in width. Waterproofing and response properties were checked in all transducers before implantation. The silicon-coated wires (RSF 66/0.03; Sanyo Electric Wire, Osaka, Japan) from the transducer were exteriorized through the abdominal wall and passed under the skin toward the back of the neck. Truncal subdiaphragmatic vagotomy was then performed. The lower part of the esophagus was exposed, and the dorsal and ventral vagus nerves were isolated. Both branches of the vagus nerves were cut, and segments of approximately 3 mm in length were resected. All the neural connections in the resected area were peeled completely using a wiping tissue (Kimwipes; Nippon Paper Crecia, Tokyo, Japan). Complete disconnection was confirmed under a stereomicroscope. The anatomy of the vagus nerve has been described in Biology of *Suncus* [33]. In sham-operated suncus, the vagus nerves were exposed but not cut. An intravenous catheter was inserted into the right jugular vein and also exteriorized to the back. The catheter was filled with heparinized saline (100 units/mL) to prevent coagulation. Two days after surgery, interdigestive gastric motility was recorded. After performing the experiment, the suncus were anatomized and confirmed the location of the transducer and the completion of vagotomy. A total of 23 animals were used in this study. Of these 23 animals, 12 received a sham operation. All sham operations were successful. Another 11 animals underwent vagotomy, and the operation could not be completed in only 1 animal. We excluded that animal from further experimentation.

### Administration of motilin and ghrelin in the fasted and fed state

The administration of motilin was initiated 10 min after the completion of spontaneous phase III contractions in the fasted suncus. Synthetic suncus motilin was administered as a continuous infusion at doses of 50 ng·kg^−1^·min^−1^ for 10 min; the continuous intravenous. infusion volume was 50 µL·100 g BW^−1^·min^−1^. The administration of ghrelin was initiated 10 min after the initiation of spontaneous phase II contractions in the fasted suncus. Acyl ghrelin was administered as a continuous infusion at doses of 0.1, 0.3, 1, 3, and 10 µg·kg^−1^·min^−1^ for 10 min. To observe postprandial contractions, the animals were given 1 g of food (trout pellets) during phase I. The administration of motilin was initiated 20 min after feeding, and was provided as a continuous infusion at doses of 50 ng·kg^−1^·min^−1^ for 10 min. The interval between each administration was defined as a minimum of 1 cycle of the MMC.

### Monitoring of gastric motility

The analog signals were amplified by an amplifier that was developed in the workshop at Saitama University. The amplifier provides up to 9000-fold amplification with a treble cutoff frequency of approximately 1.5 Hz. The amplified signals were then converted by an analog–digital converter (ADC-20, Pico Technology Ltd, St Neots, UK), and the digital signals were recorded by the software (PicoLog, Pico Technology Ltd, St Neots, UK) with a sampling interval of 100 ms. Gastric contraction data were processed with a frequency cutoff of 0.8 Hz using software (Chart 5; ADInstruments, Dunedin, New Zealand). Data collection began at 10:00 and stopped at 18:00 or 20:00. Suncus were fasted during the data collection period, except for those in the postprandial experiment. The interval between each experiment was defined as a minimum of 1 cycle of the MMC. Phase III contractions of MMC in conscious suncus were defined on the basis of those in dogs and humans (i.e., clustered contractions with an amplitude of >8 g that lasts for >5 min). Phase I was defined as the period of motor quiescence with contractions of an amplitude of <0.5 g. Phase II was defined as the irregular contractions that followed phase I and had an amplitude of >3-fold those in phase I.

### Data and statistical analysis

Gastric motility was quantified using the motility index (MI). In this study, the MI during a 10-min drug administration was defined as the percentage of the area under the curve, which is equivalent to the integrated area between the contractile wave and the baseline, for the 10-min duration of adjacent phase III contractions. Values were recorded as mean ± standard error. Statistical analysis was performed using Student's *t*-test. A p-value of <0.05 was considered statistically significant.

## Results

### Spontaneous gastric contractions in the fasted state

**Figure 1:**
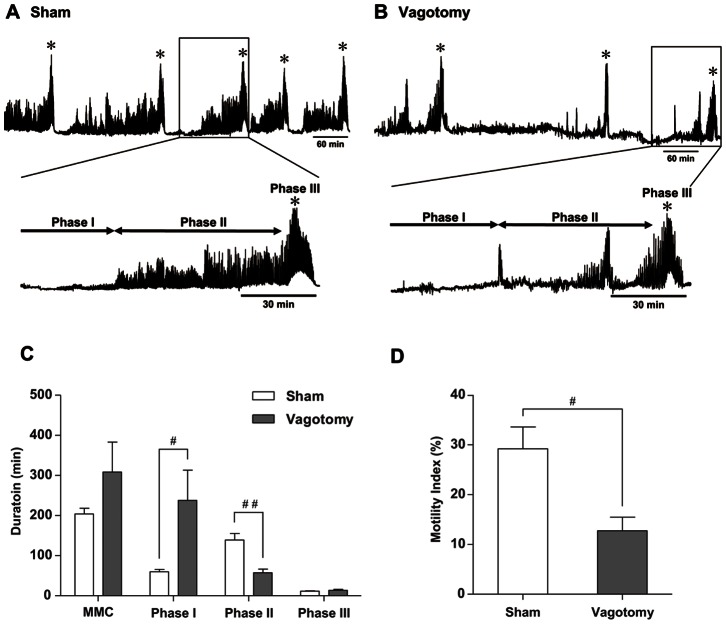
Spontaneous gastric contractions in sham-operated and vagotomized, fasted suncus. (A) Regular, cyclic, spontaneous MMC with phase I, II, and III contractions was observed in the stomach of sham-operated suncus. (B) In vagotomized suncus, the amplitude of phase II contractions was decreased, although spontaneous MMC was also observed. Asterisks indicate the phase III contraction. (C) The entire period of the MMC and the duration of phase III were unchanged between sham and vagotomized suncus; however, the duration of phase I was increased in vagotomized suncus. In contrast, the duration of phase II was significantly shorter in vagotomized suncus than in sham-operated animals. (D) The motility index of phase II was significantly lower in vagotomized suncus than in sham-operated suncus. * Phase III contractions. #, p<0.05; ##, p<0.01; n = 5.

In the fasted state, cyclic MMC comprising phases I, II, and III was clearly observed in both sham-operated and vagotomized suncus (Figs. 1A and B). However, the duration of phase II in vagotomized suncus (57±9 min) was significantly shorter than that of sham-operated suncus (139±16 min). The duration of phase I in vagotomized suncus was increased (238±76 min vs. 60±6 min). The entire period of the MMC and the duration of phase III were not significantly different between sham-operated and vagotomized animals (Fig. 1C). The phase II MI in vagotomized suncus (13%±3%) was significantly lower than that of sham-operated animals (29%±5%) (Fig. 1D).

### Effect of ghrelin on phase II of the MMC

**Figure 2:**
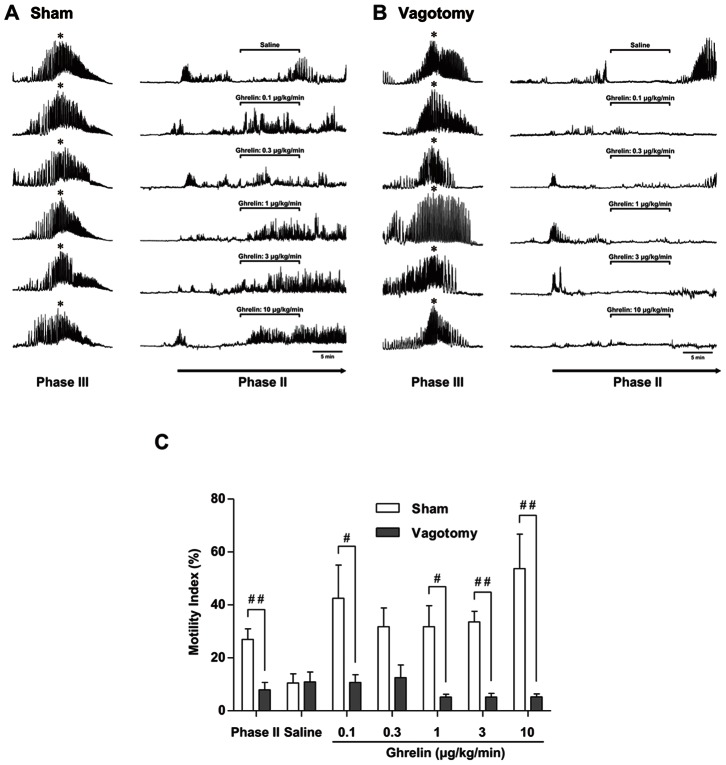
The effect of ghrelin administration on phase II of the MMC. Ghrelin (0.1, 0.3, 1, 3, or 10 µg·kg^−1^·min^−1^ for 10 min) was intravenously administered during phase II of the MMC (10 min after the initiation of phase II) in sham-operated (A) and vagotomized (B) suncus. Ghrelin administration was found to enhance the phase II contraction in sham-operated suncus but not vagotomized suncus. (C) The motility index was statistically increased in sham-operated suncus compared with vagotomized suncus. * Phase III contractions. #, p<0.05; ##, p<0.01; n = 4.

At 10 min after the initiation of phase II contractions, saline or ghrelin (0.1, 0.3, 1, 3, or 10 µg·kg^−1^·min^−1^) was intravenously infused for 10 min. In sham-operated suncus, ghrelin increased the amplitude of spontaneous phase II contractions (Fig. 2A). However, ghrelin did not alter the amplitude of spontaneous phase II contractions in vagotomized suncus (Fig. 2B). The MI during ghrelin administration in sham-operated suncus was significantly higher than that in vagotomized animals (Fig. 2C).

### Effect of motilin on phase I of the MMC

**Figure 3:**
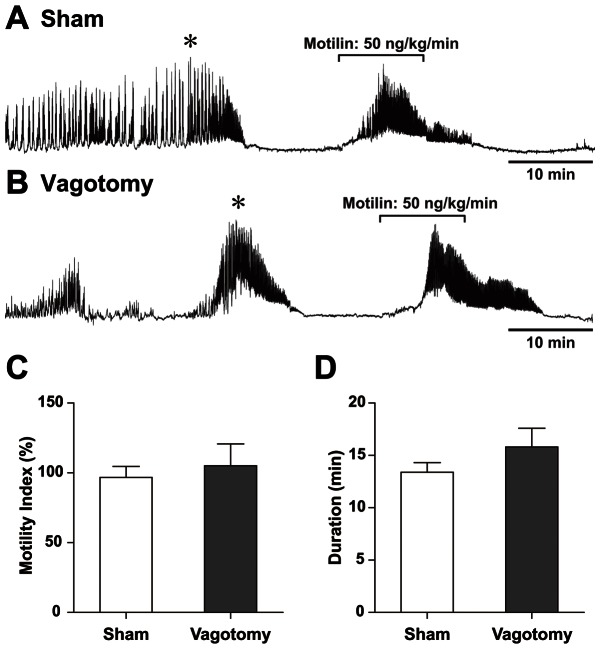
The effect of motilin administration on phase I of the MMC. Motilin (50 ng·kg^−1^·min^−1^ for 10 min) was intravenously administered during phase I of the MMC (10 min after the end of phase III) in sham-operated (A) and vagotomized (B) suncus. The motility index (C) and duration (D) of motilin administration was not differentially affected between the stomachs of sham-operated and vagotomized suncus. * Phase III contractions. Sham, n = 8; vagotomy, n = 5.

Ten minutes after the completion of spontaneous phase III contractions, motilin (50 ng·kg^−1^·min^−1^) was intravenously injected for 10 min. Motilin-induced phase III–like gastric contractions were observed in both sham-operated and vagotomized suncus (Fig. 3A and 3B). The MI during motilin administration, and the duration of the motilin-induced contractions, were not significantly different (Figs. 3C and D).

### Postprandial gastric motility and the effect of motilin in the postprandial state

**Figure 4:**
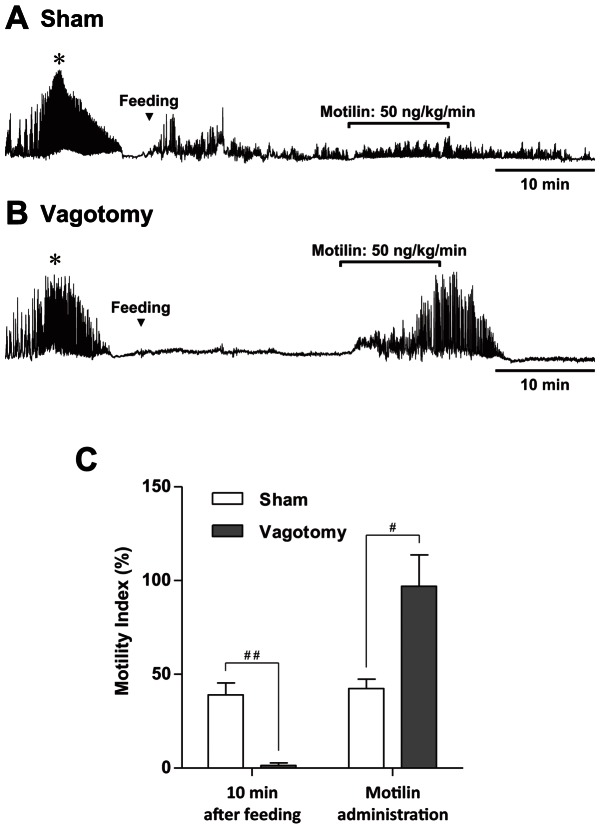
The effect of motilin administration in the postprandial state. Motilin (50 ng·kg^−1^·min^−1^ for 10 min) was intravenously administered in the postprandial state (20 min after the initiation of feeding) in sham (A) and vagotomized (B) suncus. Motilin did not induce contraction in the stomachs of sham-operated animals (A); however, motilin induced strong contractions in the stomachs of vagotomized suncus (B). The motility index for 10 min from feeding was significantly decreased in vagotomized suncus (C). However, the motility index during motilin administration increased in vagotomized suncus (D). * Phase III contractions. #, p<0.05; ##, p<0.01; sham, n = 5; vagotomy, n = 3.

After feeding, phase I of the MMC was replaced by noncyclical postprandial contractions in sham-operated suncus. A 10-min administration of motilin (50 ng·kg^−1^·min^−1^) did not induce phase III–like contractions (Fig. 4A). In contrast, feeding did not interrupt phase I of the MMC in vagotomized suncus, and exogenous motilin induced strong phase III–like contractions as in the fasted state (Fig. 4B). The MI for 10 min after the initiation of feeding in vagotomized suncus was significantly lower than that in the sham-operated group. The MI of motilin-induced contractions in the postprandial period in vagotomized animals was significantly higher than that of sham-operated suncus (Fig. 4C).

## Discussion

The mechanisms of controlling the MMC are of great interest, and the MMC is important for maintaining quality of life (QOL). It is reported that the absence of an effective MMC leads to intraluminal bacterial overgrowth in the small intestine [34], which causes chronic diarrhea, weight loss, and malabsorption. Phase III MMC contractions are believed to be induced by elevated motilin levels [32], [35]. However, many recent reports claim that ghrelin is also closely associated with GI motility. Ghrelin stimulates gastric phase III–like contractions in rats and mice [18], [19]. In humans, ghrelin induces premature phase III contractions [36]. Thus, the MMC is believed to be coordinately controlled by endogenous motilin and ghrelin. In addition to these hormones, autonomic nerves—particularly the vagus nerve—should be investigated with respect to the regulation of the MMC. However, as described above, research addressing the involvement of the vagus nerve in the MMC in humans and dogs has been inconclusive.

Ghrelin is believed to exert its effect on gastric motility through 2 pathways—the peripheral and central nervous systems. In our previous in vitro study, ghrelin administration with pretreatment of a low dose of motilin induced gastric contraction in a dose-dependent manner [37], indicating that ghrelin can act on peripheral sites. On the other hand, ghrelin induced gastric contractions via the vagus nerve in rats and mice [18], [19]. Ghrelin was also found to induce gastric motility in anaesthetized guinea pigs through the activation of vagovagal reflex pathways [38]. In the present study, cyclic MMC consisting of phase I, II, and III was observed in both sham-operated and vagotomized suncus in the fasted state, suggesting that the vagus nerve is not essential for the occurrence of the MMC. However, the duration and MI of spontaneous phase II contractions were significantly shortened and decreased in vagotomized suncus compared with those of sham-operated suncus. In our previous report, we indicated that the administration of ghrelin in the latter half of phase I and during phase II stimulated gastric contractions, and continuous infusion of a ghrelin antagonist (D-Lys3-GHRP6) suppressed phase II contractions [37]. This suggests that ghrelin is essential for initiating and maintaining phase II contractions. Moreover, in the present study, we found that the subdiaphragmatic vagotomy significantly attenuated not only motility, but also the stimulatory effect of ghrelin (0.1–10 µg·kg^−1^·min^−1^) in the phase II period, and ghrelin did not enhance phase II contractions in vagotomized suncus. These results strongly suggest that ghrelin acts directly on vagal afferent pathways or on the central nervous system, and then induces gastric phase II contractions through the vagus nerve. However, it could be possible that ghrelin and the vagus nerve induce phase II contractions through a separate pathway.

As described above, the vagus nerve is essential for inducing gastric phase III–like contractions in rodents; however, in dogs, the relationship between the vagus nerve and phase III contractions is unclear. Motilin may induce GI contraction through 3 pathways: the autonomic nervous system, myenteric neurons, or smooth muscle [3]–[10], [39]. However, the detailed mechanisms of motilin-induced contractions remain obscure. In this study, motilin evoked similar gastric contractions in sham-operated and vagotomized suncus, and our previous in vitro study demonstrated that an isolated whole suncus stomach responded to motilin in a tetrodotoxin- and atropine-sensitive manner [40]. This indicates that motilin-induced suncus gastric contractions are mediated by the myenteric cholinergic neural pathway and not the vagus nerve. We previously showed that not only a motilin antagonist (MA-2029), but also a ghrelin antagonist (D-Lys3-GHRP6), delayed the occurrence of phase III contractions [37]. This suggests that the coordination of motilin and ghrelin are necessary to initiate phase III contractions. As discussed above, the stimulatory effect of ghrelin in phase II contractions is believed to be mediated by the vagus nerve; however, spontaneous phase III contractions were observed even in vagotomized suncus. These results suggest that ghrelin influences phase III contractions independently of the vagus nerve. We previously reported that despite the observation that the isolated whole suncus stomach did not respond to a high dose of ghrelin (10^−7^ M), ghrelin (10^−10^ to 10^−7^ M) induced gastric contractions in a dose-dependent manner after pretreatment with a low dose of motilin (10^−10^ M) that was unable to stimulate whole stomach contractions alone. The final mediator of this synergistic effect was found to be cholinergic neurons [37]. The plasma concentration of ghrelin is known to correlate with MMC-like motilin. In a canine study, the peak plasma ghrelin level was observed 20–25 min after the peak plasma motilin level [41]. Together, these results suggest that increased blood ghrelin in the fasted state may act on ghrelin receptors in the stomach and interact with nerve excitation provoked by motilin, leading to spontaneous phase III contractions.

The fasted state is changed by feeding, and the irregular, continuous motor activity of postprandial contractions is induced. Several studies on dogs have demonstrated a potential role for the vagus nerve in the initiation of postprandial contractions. Specifically, after vagotomy, the initiation of the feeding pattern requires more food [42], and the duration of the MMC disruption is reduced [43]. Additionally, vagal blockage by bilateral cervical cooling abolishes postprandial activity [44] and attenuates solid gastric emptying [16]. The present result, that postprandial contractions did not occur after feeding in vagotomized suncus, supports these results.

**Figure 5:**
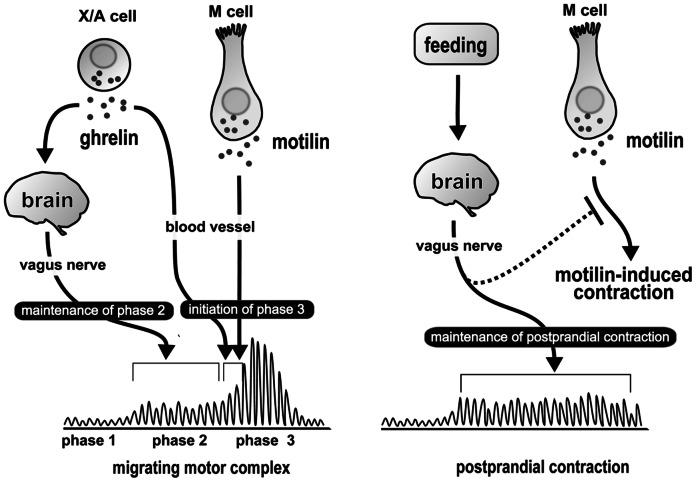
Working hypothesis of the regulatory mechanisms of the MMC and postprandial contractions in suncus. During the fasted state, endogenous ghrelin secreted from X/A cells acts on the brain and stimulates and maintains gastric phase II of the MMC through vagal efferent nerves. Ghrelin also directly acts on the stomach through the circulation, and initiates gastric phase III contractions in coordination with motilin secreted from M cells (A). Postprandial contractions are initiated and maintained by the vagus nerve, and motilin-induced contractions are inhibited by a vagus nerve–related pathway (B).

In summary, we investigated the relationship between the vagal pathway and interdigestive and postprandial contractions, and found that the ghrelin-mediated vagal pathway is important for the regulation of MMC phase II, but not for phase III contractions. Ghrelin is secreted from gastric X/A cells and stimulates phase II contractions via vagal efferents. Motilin is secreted from duodenal M cells and induces phase III contractions in coordination with ghrelin (Fig. 5A). In addition, the vagus nerve is essential for initiating the contractions in the postprandial state and is involved in the inhibition of motilin-induced contractions (Fig. 5B).
